# T2 mapping of the peritumoral infiltration zone of glioblastoma and anaplastic astrocytoma

**DOI:** 10.1177/1971400921989325

**Published:** 2021-02-11

**Authors:** Timo Alexander Auer, Maike Kern, Uli Fehrenbach, Yasemin Tanyldizi, Martin Misch, Edzard Wiener

**Affiliations:** 1 Department of Radiology, Charité University Hospital, Berlin, Germany; 2 Departement for Neuroradiology, Charite – University Hospital Berlin, Berlin, Germany; 3 Department of Neuroradiology, University Medical Center of the Johannes Gutenberg-University Mainz, Mainz, Germany; 4 Department of Neurosurgery, Charité University Hospital, Berlin, Germany

**Keywords:** Glioblastoma, glioma, MRI (magnetic resonance imaging), multiparametric imaging, T2 mapping

## Abstract

**Purpose:**

To characterise peritumoral zones in glioblastoma and anaplastic astrocytoma evaluating T2 values using T2 mapping sequences.

**Materials and methods:**

In this study, 41 patients with histopathologically confirmed World Health Organization high grade gliomas and preoperative magnetic resonance imaging examinations were retrospectively identified and enrolled. High grade gliomas were differentiated: (a) by grade, glioblastoma versus anaplastic astrocytoma; and (b) by isocitrate dehydrogenase mutational state, mutated versus wildtype. T2 map relaxation times were assessed from the tumour centre to peritumoral zones by means of a region of interest and calculated pixelwise by using a fit model.

**Results:**

Significant differences between T2 values evaluated from the tumour centre to the peritumoral zone were found between glioblastoma and anaplastic astrocytoma, showing a higher decrease in signal intensity (T2 value) from tumour centre to periphery for glioblastoma (*P = *0.0049 – fit-model: glioblastoma –25.02± 19.89 (–54–10); anaplastic astrocytoma –5.57±22.94 (–51–47)). Similar results were found when the cohort was subdivided by their isocitrate dehydrogenase profile, showing an increased drawdown from tumour centre to periphery for wildtype in comparison to mutated isocitrate dehydrogenase (*P* = 0.0430 – fit model: isocitrate dehydrogenase wildtype –10.35±16.20 (–51) – 0; isocitrate dehydrogenase mutated 12.14±21.24 (–15–47)). A strong statistical proof for both subgroup analyses (*P* = 0.9987 – glioblastoma *R*^2^ 0.93±0.08; anaplastic astrocytoma *R*^2^ 0.94±0.15) was found.

**Conclusion:**

Peritumoral T2 mapping relaxation time tissue behaviour of glioblastoma differs from anaplastic astrocytoma. Significant differences in T2 values, using T2 mapping relaxation time, were found between glioblastoma and anaplastic astrocytoma, capturing the tumour centre to the peritumoral zone. A similar curve progression from tumour centre to peritumoral zone was found for isocitrate dehydrogenase wildtype high grade gliomas in comparison to isocitrate dehydrogenase mutated high grade gliomas. This finding is in accordance with the biologically more aggressive behaviour of isocitrate dehydrogenase wildtype in comparison to isocitrate dehydrogenase mutated high grade gliomas. These results emphasize the potential of mapping techniques to reflect the tissue composition of high grade gliomas.

## Introduction

Glioblastomas (GBMs) and anaplastic astrocytomas (AA3s) represent the majority of high grade gliomas (HGGs) and are the most common malignant adult brain tumours. For treatment, prediction and prognostic decision HGGs have to be classified. The gold standard therefore is the World Health Organization (WHO) classification. Within this classification, HGGs are categorised according to the histological morphology and since the last update in 2016 to genotypic parameters as they also have an impact on predicting the outcome. AA3s are categorised as WHO grade III and GBMs as WHO grade IV tumours.^1,2^ Especially in HGGs, isocitrate dehydrogenase (IDH) seems to have the most crucial impact on prognosis. Furthermore, IDH mutation helps to distinguish between primary and secondary GBMs, as the mutation is rarely seen in de novo GBMs.^2^

Within GBMs, another well-known molecular parameter with a reported predictive value is the methylation of the methylguanine methyltransferase (MGMT) promotor gene. Patients with a methylated MGMT promotor benefit from adjuvant chemotherapy.^2,3^ As a reaction to these findings, the WHO classification included the genotypic parameters in the revised version from 2016.^4,5^

The management of such highly aggressive tumours as GBMs is difficult. The development of reliable and easy to realise response criteria is challenging. To date, the gold standard for response assessment is the response assessment in neuro-oncology (RANO) criteria.^6^ However, the detection of any relapse situation after resection remains difficult. The reason for this might be that the majority of recurring HGGs is within 2–3 cm of the resection margin. This aspect reflects the importance of the peritumoral zone, which is the active spot of tumour growth and response.^7^ With this the preoperative visualisation of the infiltrative peritumoral zone is crucial for planning the resection margin and subsequently directly affecting the frequency of margin recurrences.^8^ The major problem with this zone is that the peritumoral zone may represent fluid-filled compartments due to the disruption of the blood–brain barrier (BBB) and/or accumulation of active tumour cells.^9^ According to Lemee et al. the peritumoral zone is radiologically defined as the healthy, magnetic resonance non-enhancing brain area a few centimetres around the tumour. It is usually T2-hyperintense, representing vasogenic oedema, which may indicate an infiltration of tumour cells.^10^ To date it is not possible to distinguish between peritumoral oedema or infiltration of tumour cells by using the ‘established’ magnetic resonance imaging (MRI) features. No ‘newer’ MRI technique (diffusion weighted imaging (DWI) and apparent diffusion coefficient (ADC), susceptibility weighted imaging (SWI), dynamic contrast enhancement (DCE), spectroscopy, etc.) enables a differentiation sufficiently. Furthermore, this zone is of great interest, regarding the different aggressive behaviour in tumours within the same WHO grade. It has been described elsewhere that isocitrate dehydrogenase wildtype (IDHw) AA3 HGGs show a more aggressive behaviour than isocitrate dehydrogenase mutated (IDHm) AA3 HGGs.^2^ This might originate from more active tumour cells and with this theoretically directly influencing the resection margin. If IDHw are treated similarly to IDHm AA3 in accordance with their WHO grade, early recurrence might increase.

Advanced MRI mapping techniques quantify the relaxation time at different echo times pixelwise and are able to give more accurate information about tissue structure and composition. Usually the magnetic resonance image contrast is determined by the specific T2 relaxation times. Oedema, tumour infiltration and an increased blood flow or water diffusion processes are known to influence the image contrast in T2-weighted images. A quantitative approach measuring the T2 relaxation times as absolute numbers in milliseconds (ms) and coding them colour or gray-scaled, also known as T2 maps, seems to be the most reliable, objective and reproducible imaging technique to visualise the image contrast in T2-weighted/fluid-attended inversion recovery (FLAIR) images.^11–14^ This might have the potential to distinguish between tumour cells and vasogenic oedema in the peritumoral zone. Recent studies report an association between T2 mapping relaxation times and the molecular background in gliomas.^15^

The purpose of this study was to characterise and gain more insights into the peritumoral zones of GBMs and AA3s according to their IDH profiles. Therefore, T2 values have been evaluated, at different echo times using T2 mapping sequences.

## Materials and methods

### Patients

A total of 41 patients with either singular GBM or AA3 were enrolled from April 2015 to December 2016. Patients younger than 18 years or with prior surgery were excluded. Patients were divided into a GBM (*n* = 22) and a AA3 (*n* = 19) cohort. Histopathological reports were available for all patients and IDH status was determined by immunohistochemistry. The study protocol (ethical application number EA1/306/16) conforms to the ethical guidelines of the 1975 Declaration of Helsinki. Some of the patients included in the present analysis participated in a previous study.^15^

### Imaging

MRI was performed on a 1.5T (Avanto Magnetom; Siemens, Erlangen, Germany) or on a 3T scanner (Skyra; Siemens, Erlangen, Germany). MRI of the brain consisted of an axial T1-weighted sequence, repetition time (TR) 550 ms, echo time (TE) 8.9 ms, slice thickness 5 mm; in-plane resolution 0.8984 mm × 0.8984 mm, acquisition matrix 256 × 216), T2 fat saturation (T2-fs) axial (TR 4000 ms, TE 92 ms, slice thickness 3 mm, field of view (FOV) 186 × 230 rows, in-plane resolution 0.4492 mm × 0.4492 mm), axial FLAIR sequence (TR 8000 ms, TE 84 ms, slice thickness 4 mm, acquisition matrix 320 × 210; in-plane resolution 0.7188 mm × 0.7188 mm), T2 mapping (TR 3100 ms, TE 13.8–165.6 ms with 12 TEs: 13.8 ms, 27.6 ms, 41.4 ms, 55.2 ms, 69 ms, 82.2 ms, 96.6 ms, 110.4 ms, 124.2 ms, 138 ms, 151.8 ms, 165.6 ms) and magnetisation-prepared rapid gradient echo (MPRAGE) sequence post contrast (TR 2200 ms, TE 2.67 ms, slice thickness 1 mm, inversion time 900 ms, in-plane resolution 0.9766 × 0.9766 mm, acquisition matrix 256 × 246). T2 maps were reconstructed by using a voxelwise, monoexponential non-negative least-squares fit analysis (MapIt; Siemens, Erlangen, Germany) with a voxel size of 1.9 × 1.0 × 3 mm³.

### Image processing and analysis

**Figure 1.**
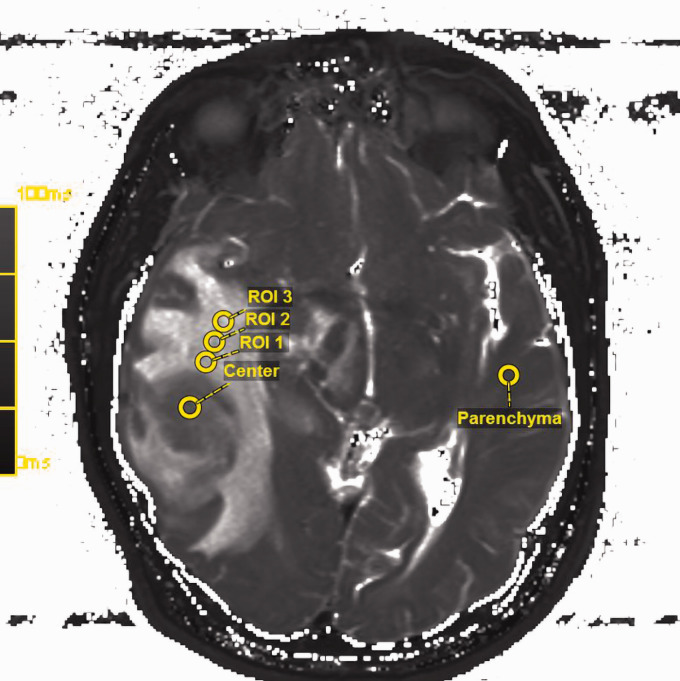
Region of interest (ROI) placement in the raw T2 map in accordance with the fluid-attended inversion recovery (FLAIR) sequences. The slice with the largest diameter of peritumoral T2-weighted/FLAIR hyperintensity was chosen, the anatomical centre of the tumour was delineated. Another ROI was placed in the healthy- appearing white matter of the contralateral lobe using an image processing program to ensure reliability of the measurements. The spatial T2 value distribution across the tumour radius was measured at four different locations from the tumour centre to the outer infiltration zone by the placement of three further ROIs.

Images were postprocessed with the visage software tool (Visage Imaging/Pro Medicus Ltd., version 7.1.10). T2 map ROIs were drawn manually by an experienced neuroradiologist (>10 years of experience) plus a second reader (2 years of experience) with a standardised diameter of 5 mm in one representable slice. The ROIs were placed radiating out from the centre of the tumour in one line out into the peritumoral zone/oedema with a maximum of three ROIs around the tumour. The ROIs covered representable tissue of a peritumoral zone, while areas of necrosis and vessels were excluded (MK, EW). The slice with the largest diameter of peritumoral T2-weighted/FLAIR hyperintensity was chosen, the anatomical centre of the tumour was delineated. Another ROI was placed in the healthy-appearing white matter of the contralateral lobe using an image processing program to ensure reliability of the measurements (Figure 1).

### Statistical analysis

The data were analysed by using XLSTAT, version 2011.3.01 (Addinsoft SARL, New York, NY, USA). The Mann–Whitney U-test was used as a two-tailed test to compare each group’s median. P values of less than 0.05 were regarded as statistically significant. The spatial T2 value distribution across the tumour radius at four different locations from the tumour centre to the outer infiltration zone adjacent to healthy brain tissue was analysed based on the ROI evaluation. Mean T2 values obtained from these ROIs were modelled using a second-degree polynomial function. The coefficients of the polynomial were calculated by minimising the sum of the squares of the deviations of the data from the model. Both the original data and the model data were presented in the plots. The second-degree fit accurately follows the basic shape of the data. Higher-degree polynomial fitting does not improve the results of the model. A higher fit model value (positive or negative) describes the curve’s opening angle. To validate the model the coefficient of determination (*R*^2^) with an intercept range between 0 and 1 was calculated. The stretching factor of the second-degree fit is a measure of the increase of T2 across the tumour and was evaluated in every subject and presented in the model function. The coefficients of determination and stretching factors for both groups were compared using the Mann–Whitney U-test.

## Results

### T2 mapping analyses

#### GBM versus AA3

**Figure 2.**
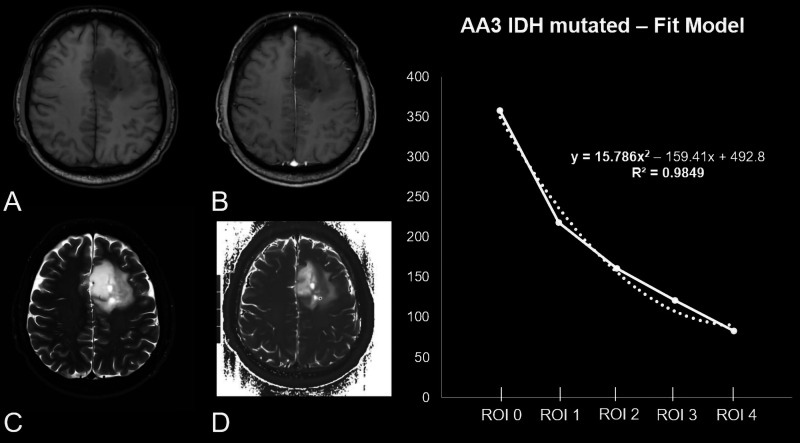
Patient, 49 years old, with a non-contrast enhanced (CE) T1-weighted heterogeneous-appearing anaplastic astrocytoma in the left frontal lobe (a) without a significant positive CE in the T1 CE sequence (b). (c) Unilateral surrounding oedema in the fluid-attended inversion recovery sequence. (d) The original raw T2 map. The diagram shows the curve progression from tumour centre to periphery calculated by means of a fit model. As reflected by 15.786x^2^ the curve’s opening angle is positive.

**Figure 3.**
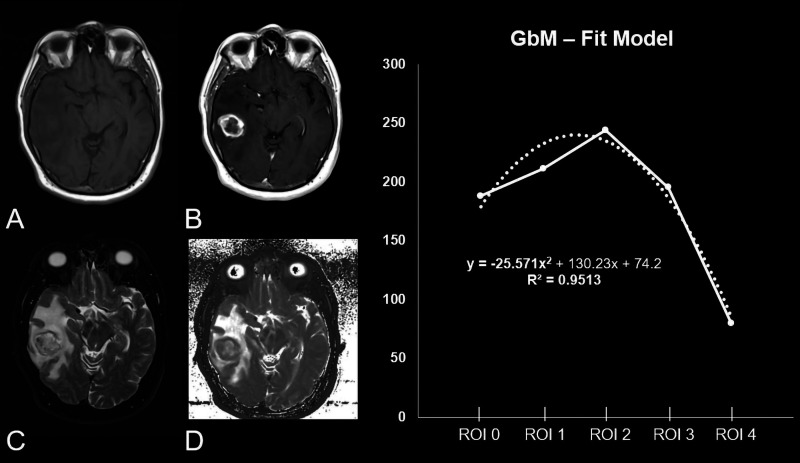
Patient, 63 years old, with a non-contrast enhanced (CE) T1-weighted heterogeneous glioblastoma in the right temporal lobe (a) and a significant positive CE in the T1 CE sequence (b). (c) A large perifocal oedema in the fluid-attended inversion recovery sequence. (d) The original raw T2 map. The diagram shows the curve progression from tumour centre to periphery calculated by means of a fit model. While the *y*-axis describes the median T2 values originating out of the T2 map; the *x*-axis shows the different regions of interest (ROIs) which were placed as follows: 1 – ROI 0: inside the tumour centre; 2 – ROI 1: 0–5 mm of the peritumoral oedema/zone; 3 – ROI 2: 5–10 mm of the peritumoral oedema/zone; 4 – ROI 3: 10–15 mm of the peritumoral oedema/zone and 5 – ROI within healthy brain parenchyma on the contralateral side. As reflected by −25.571x^2^ the curve’s opening angle is negative.

**Figure 4.**
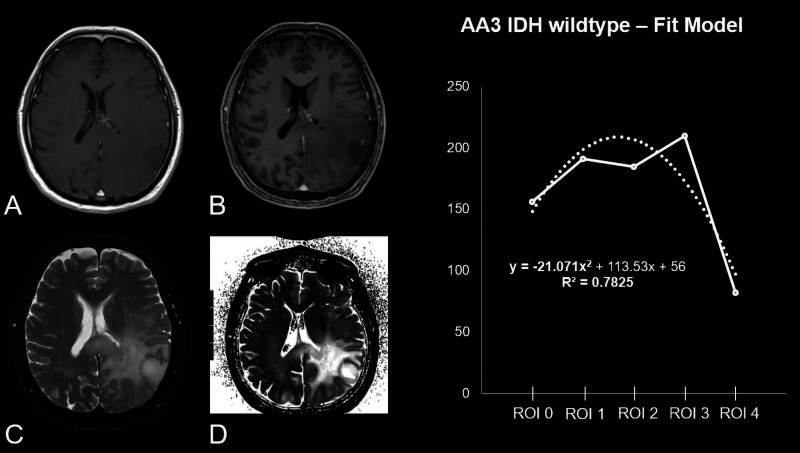
Patient, 63 years old, with a non-contrast enhanced (CE) T1-weighted homogeneous-appearing anaplastic astrocytoma isocitrate dehydrogenase wildtype in the left parietal lobe (a) with marginal CE in the T1 CE sequence (b). (c) Surrounding oedema in the fluid-attended inversion recovery sequence. (d) The original raw T2 map. The diagram shows the curve progression from tumour centre to periphery calculated by means of a fit model. As reflected by −21.071x^2^ the curve’s opening angle is negative.

Our calculated model parameter out of the T2 relaxation times showed a significantly different curve progression from tumour centre to periphery in GBM when compared to AA3 (*P* = 0.0049 – fit model (curves skewness): GBM –25.02±19.89 ((–54)–10); AA3 –5.57±22.94 ((–51)–47)). The *R*^2^ fit model showed a strong statistical proof for both subgroup analyses (*P*  =  0.9987 – GBM *R*^2^ 0.93±0.08; AA3 *R*^2^ 0.94±0.15) (Table 1, Figures 2, 3 and 4).

**Table 1.** Patient characteristics.

	AA3 (*n*=19)	GBM (*n*=22)	*P* value
Age (mean, range)	43.11±11.22 (29–63)	62.14±14.71 (34–83)	<0.001*/<0.001
IDH mutated	38.55±8.71 (29–54)	39±0 (39)	
IDH wildtype	49.38±11.72 (34–63)	63.24±14.11 (34–83)	
Sex (no.)			0.184*/0.537
Female	9 (47.37%)	8 (36.36%)	
Male	10 (52.63%)	14 (63.64%)	
IDH			<0.001
Mutated	11 (57.89%)	1 (4.55%)	
Wildtype	8 (42.22%)	21 (95.45%)	
MGMT			0.001*/0.052
Mutated	13 (68.42%)	9 (40.91%)	
Wildtype	4 (21.05%)	12 (54.55%)	
Not determined	2 (10.53%)	1 (4.55%)	
Localisation			0.061†*/0.510†
	Frontal (26.32%) Parietal (15.79%) Fronto-parieto-temporal (15.79%) Temporal (10.53%) Fronto-temporal (10.53%) Frontoparietal (5.26%) Parieto-temporal (5.26%) Multilobar (5.26%) Thalamic (5.26%)	Temporal (27.27%) Frontal (18.18%) Frontoparietal (18.18%) Bifrontal (9.09%) Parietal (9.09%) Parieto-occipital (9.09%) Fronto-temporal (4.55%) Periventricular (4.55%)	
T1w/T1w CE diameter (mm)	43.58±20.11 (17–81)	40.91±17.16 (18–86)	0.025*/0.811
IDH mutated	54.82±18.50 ([32–81)	26±0 (26)	
IDH wildtype	28.13±8.91 (17–43)	41.62±17.25 (18–86)	
T2w/FLAIR diameter (mm)	15.84±6.14 (8–29)	20.55±6.04 (10–36)	0.011*/0.012
IDH mutated	15.09±6.77 (8–29)	14±0 (14)	
IDH wildtype	16.88±5.44 (8–27)	20.86±6.00 (10–36)	

AA3: anaplastic astrocytoma; CE: contrast enhanced; FLAIR: fluid-attenuated inversion recovery; GBM: glioblastoma; IDH: isocitrate dehydrogenase; MGMT: methylguanine methyltransferase; T2w: T2-weighted.

*This *P* value relates to the difference between the isocitrate dehydrogenase mutated and the isocitrate dehydrogenase wildtype tumours independently of the tumour grade.

†This *P* value relates to fronto-parietal or not fronto-parietal localisation.

#### IDHw versus IDHm HGG

**Figure 5.**
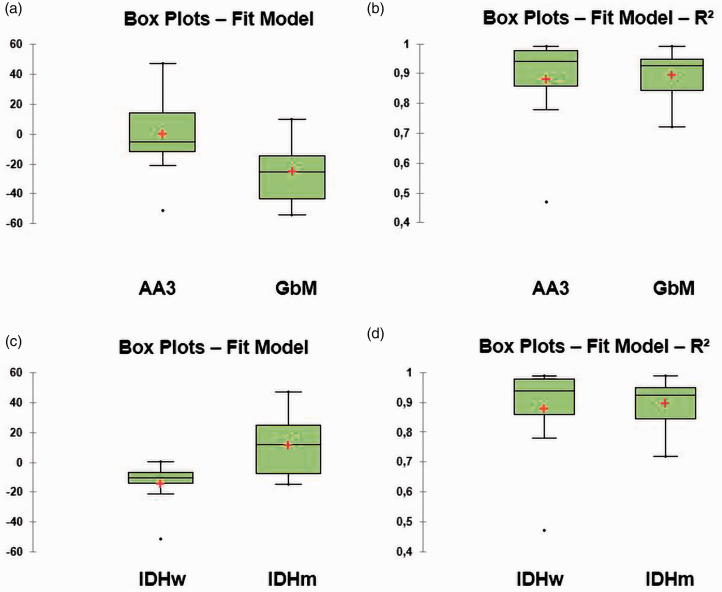
(a) Significant difference between the curve progression for the medians, when the cohort is divided into anaplastic astrocytoma and (b) illustrated with R2 a strong statistical validity for the analyses, when the cohort is subdivided by World Health Organization grade; (c) Significant difference in between curve progression for the medians, when the cohort is divided by isocitrate dehydrogenase (IDH) mutational state; (d) illustrated with R2 a strong statistical validity for the analyses, when the cohort is subdivided into IDH mutated and IDH wildtype high grade glioma.

When divided by IDH mutational status the T2 relaxation times also showed significantly different curve progression behaviour in IDHw gliomas as compared to IDHm gliomas (*P* = 0.0430 – fit model: IDHw –10.35±16.20 ((–51)–0); IDHm 12.14±21.24 ((–15)–47)). The fit model showed a fair statistical proof for both subgroup analyses divided by mutational state (*P* = 0.4180 – IDHw *R*^2^ 0.94±0.17; AA3 *R*^2^ 0.96±0.13) (Table 1, Figures 3–5).

### Patient characteristics

Overall, there is a strong difference in age concerning the IDH status (*P<0.001*) and the WHO grade (*P<0.001*). Patients with AA3 (43.11±11.22 years) are younger than patients with GBM (62.14±14.71 years), while IDHw is associated with an older age than IDHm. There were 17 female and 24 male subjects included. MGMT promotor methylation prevails significantly in IDHm gliomas (*P<0.001*). Regarding the WHO grade, no significant differences have been reported (*P = 0.052*). The tumour localisation does not correlate significantly with the WHO grade (*P = 0.510*) or the IDH status (*P = 0.061*). There was no significant difference between the maximal axial tumour diameter in the T1/T1 contrast enhanced sequences from AA3 (43.58±20.11 (range 17–81) mm) and GBM (40.91±17.16 (range 18–86) mm); *P* = 0.811). Nevertheless, GBMs (20.55±6.04 (range 10–36) mm) had a larger diameter in the T2/FLAIR sequences, potentially reflecting the oedema/infiltration zone than AA3 (15.84±6.14 (range 8–29) mm; *P = 0.012*). Assorting by the IDH status revealed that IDHm gliomas had a significantly larger tumour diameter (*P = 0.025*) but a significantly smaller oedema/infiltration zone (*P = 0.011*) than IDHw gliomas. All other patient characteristics, distributions and values are provided in Table 2.

**Table 2.** Results.

	AA3	GBM	*P* value
Fit	–5.57±22.94 ((–51)–47)	–25.02±19.89 ((–54)–10)	0.0049
ROI 0 (ms) Tumour centre	290.37±133.65 (121–497)	197.57±74.38 (109–419)	
ROI 1 (ms) Peritumoral zone 1	206.79±69.99 (104–357)	213.57±57.97 (123–306)	
ROI 2 (ms) Peritumoral zone 2	179.74±47.63 (110–297)	229.38±73.23 (112–334)	
ROI 3 (ms) Peritumoral zone 3	189.00± 66.55 (119–290)	228.07±62.18 (116–352)	
ROI 4 (ms) Contralateral: normal brain tissue	82.72±3.50 (77–90)	84.81±3.84 (79–91)	
*R* ^2^	0.94±0.15 (0.47–0.99)	0.93± 0.08 (0.72–0.99)	0.9987
	IDH wildtype	IDH mutated	
Fit	–10.35±16.20 ((–51)–0)	12.14±21.24 ((–15)–47)	0.0430
ROI 0 (ms) Tumour centre	189.11±73.39 (109–419)	364.25±102.33 (155–497)	
ROI 1 (ms) Peritumoral zone 1	204.75±63.63 (109–306)	223.42±62.94 (146–357)	
ROI 2 (ms) Peritumoral zone 2	216.75±74.34 (110–334)	180.25±33.40 (141–246)	
ROI 3 (ms) Peritumoral zone 3	217.90±66.24 (116–352)	194.33±79.61 (121–279)	
ROI 4 (ms) Contralateral: normal brain tissue	84.36±3.75 (79–91)	82.99±3.73 (77–89)	
*R* ^2^	0.94±0.17 (0.47–0.99)	0.96±0.13 (0.56–0.99)	0.4180

AA3: anaplastic astrocytoma; GBM: glioblastoma; ROI: region of interest.

## Discussion

This study evaluates quantitative T2 mapping sequences for characterising the peritumoral zone of HGG. Peritumoral T2 mapping relaxation times of GBMs differ from AA3s. GBM median relaxation times showed a significantly different curve progression from tumour centre to periphery compared to AA3. Furthermore, when subdivided by their IDH profile, T2 relaxation times also differed between IDHw HGG and IDHm HGG. Surprisingly, AA3 IDHw showed a similar curve progression from tumour centre to periphery like GBM (Figures 2 and 5). The quantitative evaluation by the use of mapping sequences may be an approach to distinguish between peritumoral oedema and the peritumoral infiltration zone and with this giving the possibility to prognosticate tumour growth behaviour.

To date characterisation of the peritumoral zone of HGG, especially GBM, remains challenging. Of course, the RANO criteria reacted in 2010 by adding T2-weighted sequences into their assessment scheme to diagnose T2 progress.^6^ Peritumoral zones remain a spot of interest for radiologists, neurosurgeons and radiotherapy oncologists as the differentiation between pure oedema and the peritumoral infiltration zone is crucial. Non-enhancing oedema might contain tumour cells; however, this will not be visible on MRI and with this especially for neurosurgeons, a definitive resection margin remains challenging.^16^

The experience and evidence in the literature for HGG regarding mapping techniques is low, although during the past decade mapping techniques are becoming more and more popular within clinical routine (e.g. cardiac and musculoskeletal MRI) as it gives the opportunity to measure the tissue and its signal behaviour quantitatively.^17–22^ Nevertheless, already in 2012 Ellingson et al. used T2 mapping sequences to quantify oedema reduction in recurrent GBM treated with bevacizumab, suggesting that post-treatment T2 values correlate with the progression-free survival. This is giving evidence to the fact that T2 mapping sequences may be suitable to characterise peritumoral oedema further.^23^ A recent study published by Kern et al. (2020) showed that the relaxation times acquired by T2 mapping sequences were able to distinguish between IDHw and IDHm WHO grade II and III gliomas, with wildtypes showing lower T2 relaxation times.^15^ In another recently published study also by Kern et al. (2020), the group investigated the tumour centre and the tumour periphery showing that values decreased in the periphery.^15^ The authors assumed that T2 values may reflect the peripheral tumour cellular activity as this is the spot of growth and infiltration.^15^ The underlying study showed for GBM in comparison to AA3 an increased signal drawdown from tumour centre to periphery. According to the conclusion of Kern et al., the results of that study might indicate an increased tumour cellularity and with this an increased aggressiveness and increased occurrence of recurrence, after resection of GBMs compared to AA3s.

The WHO classification was revised in 2016 and now includes molecular marker profiles. The reason for this is that especially IDHw grade II and III gliomas can act like GBMs and have a similar dismal prognosis.^2,5^ In this study HGGs were not only subdivided into GBM and AA3, but the cohort was further subdivided regarding the mutilation state. Peritumoral zones were investigated and in line and according to the above-mentioned biologically more aggressive HGG wildtypes showed an increased drawdown from tumour centre to periphery, similar to GBMs. This may also emphasise the potential of T2 mapping sequences, for differentiating between HGG molecular subtypes and might give the possibility for assessing the risk of developing an early recurrence. To the best of our knowledge, no MRI method is established to define the peritumoral infiltration zone. The underlying data are emphasising further investigations regarding T2 mapping to define and gain more insight into the peritumoral zone. With this, the extent of tumour resection might be defined more precisely, directly influencing tumour burden and patient prognosis.^10,24,25^

An interesting study by Blystad et al. (2017) analysed the peritumoral oedema of malignant gliomas quantitatively also using T1 and T2 mapping sequences. The authors found a gradient of relaxation values in the peritumoral oedema closest to contrast-enhancing parts of the tumour and an increase of the gradients in the oedemas after contrast agent injection.^26^ Future prospective aims will be to refine the prognostication of mapping relaxation values to establish values to characterise further the peritumoral zone and to differentiate between oedema and infiltration.

### Limitations

The retrospective and non-blind study design has introduced some detection bias. Another limitation might be the relatively small patient cohort and a missing volume-based analysis, while this study used a regionally based approach. However, the results of this study are promising and novel. Furthermore, although the sample number is small the statistically calculated validity levels are sufficient. A further limitation is the use of two different scanners. Nevertheless, although two different types of scanner were used, T2 values of brain parenchyma are not affected significantly by the field strength.^27^

## Conclusion

This is one of the first studies investigating T2 mapping sequences to characterise the tumour centre and periturmoral zone within HGGs. The results show that the peritumoral relaxation behaviour differs from GBMs to those of AA3s. Interestingly, when subdivided by their molecular IDH profile, the more aggressive IDHw show a similar relaxation behaviour as GBMs. These results emphasise the potential of T2 mapping techniques to reflect the tissue composition of HGGs. With this a differentiation between active tumour cells and vasogenic oedema in the peritumoral zone might be possible. The results of this study add information to the ongoing discussion on how to facilitate more accurate resection margins potentially decreasing early recurrence.

## Conflict of interest

The author(s) declared no potential conflicts of interest with respect to the research, authorship, and/or publication of this article.
